# The uremic toxin indoxyl sulfate decreases osteocyte RANKL/OPG and increases Wnt inhibitor RNA expression that is reversed by PTH

**DOI:** 10.1093/jbmrpl/ziae136

**Published:** 2024-10-29

**Authors:** Neal X Chen, Kalisha D O’Neill, Hannah E Wilson, Shruthi Srinivasan, Lynda Bonewald, Sharon M Moe

**Affiliations:** Department of Medicine, Division of Nephrology and Indiana University School of Medicine, Indianapolis, IN 46202, United States; Department of Medicine, Division of Nephrology and Indiana University School of Medicine, Indianapolis, IN 46202, United States; Department of Anatomy, Cell Biology and Physiology Indiana University School of Medicine, Indianapolis, IN 46202, United States; Department of Medicine, Division of Nephrology and Indiana University School of Medicine, Indianapolis, IN 46202, United States; Department of Anatomy, Cell Biology and Physiology Indiana University School of Medicine, Indianapolis, IN 46202, United States; Department of Orthopaedic Surgery, Indiana University School of Medicine, Indianapolis, IN 46202, United States; Department of Medicine, Division of Nephrology and Indiana University School of Medicine, Indianapolis, IN 46202, United States; Department of Anatomy, Cell Biology and Physiology Indiana University School of Medicine, Indianapolis, IN 46202, United States

**Keywords:** indoxyl sulfate, uremic toxins, osteocytes, parathyroid hormone, bone remodeling, mineralization

## Abstract

Renal osteodystrophy (ROD) leads to increased fractures, potentially due to underlying low bone turnover in chronic kidney disease (CKD). We hypothesized that indoxyl sulfate (IS), a circulating toxin elevated in CKD and a ligand for the aryl hydrocarbon receptor (AhR), may target the osteocytes leading to bone cell uncoupling in ROD. The IDG-SW3 osteocytes were cultured for 14 days (early) and 35 days (mature osteocytes) and incubated with 500 μM of IS after dose finding studies to confirm AhR activation. Long-term incubation of IS for 14 days led to decreased expression of Tnfsf11/Tnfrsf11b ratio (RANKL/OPG), which would increase osteoclast activity, and increased expression of Wnt inhibitors Sost and Dkk1, which would decrease bone formation in addition to decreased mineralization and alkaline phosphatase (ALP) activity. When osteocytes were incubated with IS and the AhR translocation inhibitor CH223191, mineralization and ALP activity were restored. However, the Tnfsf11/Tnfrsf11b ratio and Sost, Dkk1 expression were not altered compared with IS alone, suggesting more complex signaling. In both early and mature osteocytes, co-culture with parathyroid hormone (PTH) and IS reversed the IS-induced upregulation of Sost and Dkk1, and IS enhanced the PTH-induced increase of the Tnfsf11/Tnfrsf11b ratio. Co-culture of IS with PTH additively enhanced the AhR activity assessed by *Cyp1a1* and *Cyp1b1* expression. In summary, IS in the absence of PTH increased osteocyte messenger RNA (mRNA) Wnt inhibitor expression in both early and mature osteocytes, decreased mRNA expression ofTnfsf11/Tnfrsf11b ratio and decreased mineralization in early osteocytes. These changes would lead to decreased resorption and formation resulting in low bone remodeling. These data suggest IS may be important in the underlying low turnover bone disease observed in CKD when PTH is not elevated. In addition, when PTH is elevated, IS interacts to further increase Tnfsf11/Tnfrsf11b ratio for osteoclast activity in both early and mature osteocytes, which would worsen bone resorption.

## Graphical Abstract

**Graphical Abstract:**
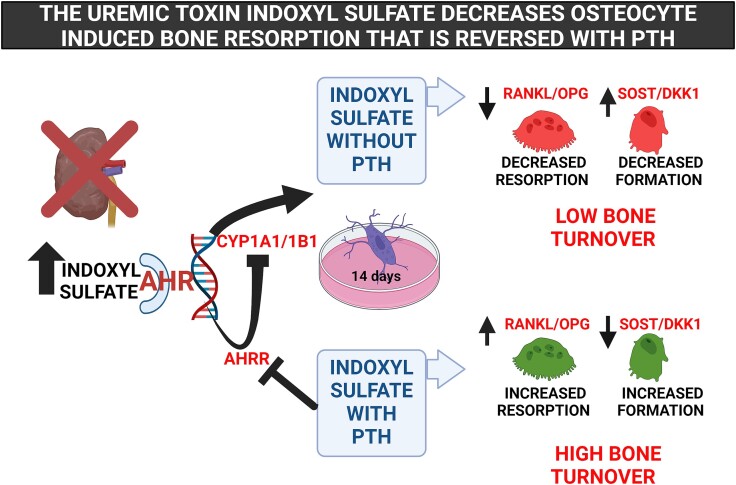

## Introduction

Chronic kidney disease (CKD) affects 15% of individuals in the United States.^1^ As CKD progresses, the risk of hip fractures increases 2- to 5-fold compared with age-matched individuals.^2^ Renal Osteodystrophy (ROD), the bone component of CKD-mineral bone disorder (CKD-MBD^3^), is traditionally assessed using histomorphometry, with high turnover bone disease treated by reducing parathyroid hormone (PTH) with calcitriol or calcimimetics. However, fracture risk is increased in CKD at extremes of both low and high PTH.^4^^,^^5^ In bone biopsy studies from humans, early CKD is associated with decreased bone formation rates and increased serum and/or bone sclerostin, which would lead to a reduction in osteocyte Wnt/ β-catenin signaling and reduced osteoblast differentiation.^6–8^ This reduction in bone formation can persist with advanced CKD and is compounded by osteoclast activation and cortical resorption when PTH is also elevated. These studies demonstrate the pathophysiology of ROD extends beyond PTH; there is an unmet clinical need to identify additional CKD-related factors that may alter osteocyte Wnt signaling.

Patients with CKD retain metabolites known as “uremic toxins” as a result of increased production and/or reduced renal excretion.^9^ Indoxyl sulfate (IS) is a gut microbially derived molecule that is sulfated in the liver and bound to albumin and is nontoxic under normal conditions. However, IS becomes a uremic toxin that accumulates in the blood of patients with CKD due to reduced renal clearance and is associated with increased all-cause mortality.^10^ IS has been shown to exert a variety of toxic effects on different cell types, including osteoblasts and osteoclasts^11–13^ and is a potent endogenous ligand for aryl hydrocarbon receptor (AhR) that is responsible for the removal of both environmental and endogenous toxins.^14^ The complexity of this signaling pathway is also demonstrated by variable bone changes in AhR null mice based on sex and animal age.^15^^,^^16^ In mice, activation of AhR with the environmental toxin 2,3,7,8-tetrachlorodibenzo-p-dioxin resulted in thinner cortical bone, altered matrix composition, and mechanically weaker bone (decreased maximal force, yield force, and energy absorption), effects reversed in AhR-/- mice^17^ indicating that AhR has a critical function in bone. This pathway may explain the environmental toxins polychlorinated biphenyls that affect teeth and bone in animals and humans.^18^ Parathyroid hormone (PTH), a key hormonal regulator of bone remodeling, is also a uremic toxin because it can be markedly elevated or inappropriately suppressed in CKD,^9^ playing a major role in the pathogenesis of CKD-MBD.

Osteocytes are critically important cells known as the “master regulator” of bone, controlling osteoblast differentiation, osteoclast resorption, and mineralization.^19^ Osteoblast differentiation to osteocytes is poorly understood, but once formed, osteocytes are long-living cells that are key to coupling bone resorption to bone formation and regulating mineralization. Osteocytes are unique in their very long lifespan,^20^ but can undergo autophagy and apoptosis leaving empty lacunae that impair connectivity between osteocytes.^19^ The osteocyte can also remove and replace their matrix to rapidly release calcium, a process called perilacunar remodeling, as occurs physiologically with lactation.^21^ When only the matrix is removed with no replacement, this is called osteocytic osteolysis as seen with hyperparathyroidism with overall bone loss.^22^ However, this matrix turnover/osteocytic osteolysis is increased in bone biopsies from patients undergoing dialysis with both high and low PTH.^23^ Similarly, we found altered matrix composition in the new periosteal and perilacunar bone in our rat model of CKD, and lowering PTH with calcimimetics only partially corrected these changes,^24^ a finding that supports factors other than PTH affecting osteocytes. A study of bone biopsies from 58 adults with various stages of CKD found increased osteocyte density and lacunar size, and decreased mineralization in the presence of high, compared with low, blood PTH levels, but lacked data from controls.^25^ Pereira et al.^26^ found evidence of osteocyte maturation failure in bone biopsies from children with CKD compared with controls. These data suggest osteocyte abnormalities may be an underlying defect in CKD, explaining the impaired osteoblast differentiation, low remodeling rates, and reduced mineralization.

In this paper, we used an osteocyte cell line IDG-SW3 that differentiates from an early to a mature osteocyte phenotype in culture with progressive mineralization, and unlike other osteocyte lines, expresses fibroblast growth factor 23 (FGF23), which is markedly elevated in CKD.^19^^,^^27^ We hypothesized that IS alters osteocyte Wnt inhibitor signaling, differentiation, and mineralization through the aryl hydrocarbon receptor. We also examined the interaction of elevated IS with and without PTH to mimic conditions observed in patients with CKD. Our results demonstrated that with short-term exposure representing temporary exposure to IS, osteocytes increased expression of inhibitors of bone formation and decreased activators of osteoclastic bone resorption temporarily leading to low bone turnover. With sustained exposure to high levels of IS, as would occur with CKD and low PTH, an enhancement of this low bone turnover phenotype was observed. However, in the presence of high PTH these effects were blunted.

## Material and methods

### Cell culture and study design

IDG-SW3 osteocytes (Immortomouse/ Dmp1-GFP-SW3) were isolated from long-bone chips from mice carrying a Dmp1 promoter driving GFP crossed with the Immortomouse, which expresses a thermolabile SV40 large T antigen regulated by interferon γ (IFN-γ)).^27^ Cells were expanded in permissive conditions (33 °C/5% CO_2_ in α-MEM with 10% fetal bovine serum, 100 units/mL of penicillin, 50 μg/mL of streptomycin, and 50 U/mL of IFN-γ, Gibco Life Technologies, Carlsbad, CA) on rat tail type 1 collagen–coated culture plates. To induce osteogenesis, cells were plated at 1 × 10^4^ cells/cm^2^ in osteogenic conditions (37 °C/8% CO_2_ with 50 μg/mL of ascorbic acid and 4 mM β-glycerophosphate in the absence of IFN-γ) in which the cells differentiate from a late osteoblast to early osteocyte (minimal mineralization) at day 14 to a mature osteocyte at day 35 as defined by expression of bone markers. Cell culture media were changed 3 times a week during the culture.

To optimize IS dosing, we first treated the cells with multiple concentrations (0, 100, 500, and 1000 μM) of IS (Sigma-Aldrich, St. Louis, MO) for 24 h in both early and mature osteocytes to confirm IS-induced AhR canonical signaling (upregulation of cytochrome P450, family 1, subfamily A, polypeptide 1 [gene *Cyp1a1*], Cytochrome P450, family 1, subfamily B [gene *Cyp1b1*], aryl hydrocarbon receptor repressor AHRR [gene *Ahrr*] expression). Based on these results, a concentration of IS of 500 μM was used in the remaining continuous long-term culture studies. To determine the mechanism by which IS affects osteocyte differentiation and gene expression, the osteocytes were treated 14 days with IS (500 μM) in the presence or absence of 10 μM AhR inhibitor CH223191 (Sigma-Aldrich, St. Louis, MO)^28^^,^^29^ and gene expression, mineralization, and AhR activation determined. To evaluate the interaction of IS with PTH we cultured osteocytes with or without IS (500 μM) in the presence or absence of PTH (100 nM; Sigma-Aldrich, St. Louis, MO, based on in vitro studies by Jilka et al.^30^) for 14 days (early osteocytes) or 35 days (mature osteocytes) and gene expression, mineralization, and AhR activation were determined.

### RNA isolation, real-time polymerase chain reaction, and cAMP measurement

Total RNA from early or mature IDG-SW3 osteocytes was isolated using miRNeasy Mini Kit (Qiagen, Germany). Gene expression of osteocyte markers were analyzed by real-time polymerase chain reaction (PCR) using Taqman gene expression assays (TaqMan MGP probes, FAM dye-labeled, Applied Biosystems, Foster City, CA) using ViiA 7 systems.^31^ The cycle number at which the amplification plot crosses the threshold was calculated (C_T_), and the ∆∆C_T_ method was used to analyze the relative changes in messenger RNA (mRNA) expression and normalized by beta-actin as previously described.^31^ Target-specific PCR primers were obtained from Applied Biosystems and included osteocyte Wnt inhibitors SOST (gene *Sost*) and Dickkopf-related protein 1 (gene *Dkk1*); osteoclast inducer receptor activator of nuclear factor kappa-Β ligand (RANKL, gene *Tnfsf11*); osteoprotegerin (OPG, gene *Tnfrsf11b*) and mineralization markers Dentin matrix acidic phosphoprotein 1 (gene *Dmp1*) and Fibroblast growth factor 23 (gene *Fgf23*). To determine the canonical signaling activity of the AhR, the gene readout of *Cyp1a1*, *Cyp1b1*, *Ahrr* were examined. Finally, PTH 1 receptor (gene *Pth1r*) gene expression was measured by PCR. For acute cAMP secretion measurement, we cultured the IDG-SW3 osteocytes with IS (500 μM) in the presence or absence of 100 nM PTH for 14 or 35 days. On day 14 or day 35, the cells were treated with IS (500 μM) in the presence or absence of 100 nM PTH for 1 h and cAMP content in conditioned media determined by immunoassay (Assay Designs).^32^

### Measurement of alkaline phosphatase activity, and mineralization in osteocytes

Alkaline phosphatase (ALP) activity was measured using *p*-nitrophenyl substrate supplied in an ALP assay kit (Pointe Scientific, Canton, MI), normalized by protein content.^33^ To assess mineralization, osteocytes were decalcified with 0.6 N HCl for 24 h and the calcium content of HCl supernatants determined colorimetrically by the *o*-cresolphthalein complexone method (Calcium kit; Pointe Scientific, Canton, MI) and normalized to protein content as previously described.^33^

### Statistics

Each experiment was repeated 3-4 times with *n* = 3 per experiment (final n of 9-12). Data were combined from the experiments and statistical analyses conducted by first excluding outliers using ROUT (Q = 1%), followed by a normality test (*p* < .05 with D'Agostino & Pearson Test). Data were log-transformed if data were not normally distributed before analyses. We used a one-way ANOVA followed by Tukey multiple comparisons test as post-hoc testing between the groups. The results are expressed as means ± standard deviation (SD) with *p* < .05 considered significant (GraphPad Prism Software version 9.4.1, La Jolla, CA).

## Results

### IS acutely and dose-dependently induced osteocyte genes responsible for inhibition of Wnt signaling and osteoclast activation

**Figure 1:**
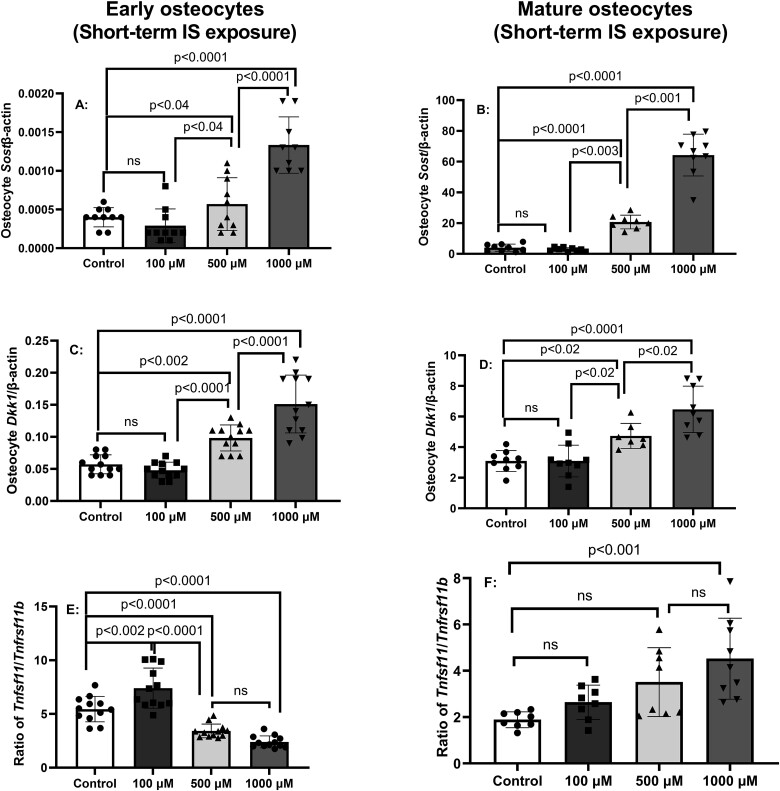
Short-term exposure to indoxyl sulfate (IS) induces messenger RNA for osteocyte genes responsible for Wnt inhibitor signaling and osteoclast activation. IDG-SW3 osteocytes were cultured for 14 or 35 days (early and mature osteocytes, respectively), then treated for 24 h with 0, 100, 500, or 1000 μM IS. The results in early and mature osteocytes show dose-dependent increase in gene expression of Sost (A and B), Dkk1(C and D), and variable effects on the gene expression of Tnfsf11/Tnfrsf11b ratio (gene for RANKL/OPG) (E and F). Data are shown as mean ± SD (*n* = 10-12). One-way ANOVA, and, if *p* < .05, Tukey multiple comparison test between groups with *p* value are shown in the graph.

To determine the dose-dependent effects of IS on osteocyte differentiation and identify an optimal dose for continuous exposure, IDG-SW3 cells were incubated with osteogenic media for 14 or 35 days, indicative of early and mature osteocytes, respectively. The cells were then treated with or without IS at various concentrations for 24 h and gene expression determined by real-time PCR. In both early and mature osteocytes, IS treatment for 24 h dose-dependently increased the expression of mRNA for the osteocyte Wnt inhibitors *Sost* (Figure 1A and B) and *Dkk1* (Figure 1C and D) at both stages of differentiation, although the *Sost* and *Dkk1*expression was greater in mature osteocytes than in early osteocytes consistent with known gene expression profiles in differentiating IDG-SW3 osteocytes.^27^ We examined the ratio of *Tnfsf11 (gene for* RANKL) mRNA expression and *Tnfrsf11b* (gene for OPG) mRNA expression to assess the ratio and potential osteoclast activation. A total of 500 or 1000 μM IS decreased the *Tnfsf11/Tnfrsf11b* ratio in early osteocyte, while only 1000 μM increased the ratio in mature osteocytes (Figure 1E and F, bottom panels). In contrast, 100 μM IS increased the expression of *Tnfsf11/Tnfrsf11b* ratio in early but not in mature osteocytes (Figure 1E and F). Thus, short-term exposure of IS dose- dependently increased osteocyte Wnt inhibitor expression and had variable effects on *Tnfsf11/Tnfrsf11b* ratio. In contrast, in early osteocytes, the short-term IS exposure only increased *Dmp1* expression at the highest dose of 1000 μM (*p* < .008, Figure S1A), and in mature osteocytes all 3 concentrations of IS similarly decreased *Dmp1* expression (*p* < .004; Figure S1B). There was no effect of IS short-term exposure on expression of *Fgf23* in early or mature osteocytes (Figure S1C and D), although the trends paralleled changes in *Dmp1*.

### IS treatment acutely and dose-dependently induced activation of AhR in early and mature osteocytes

**Figure 2:**
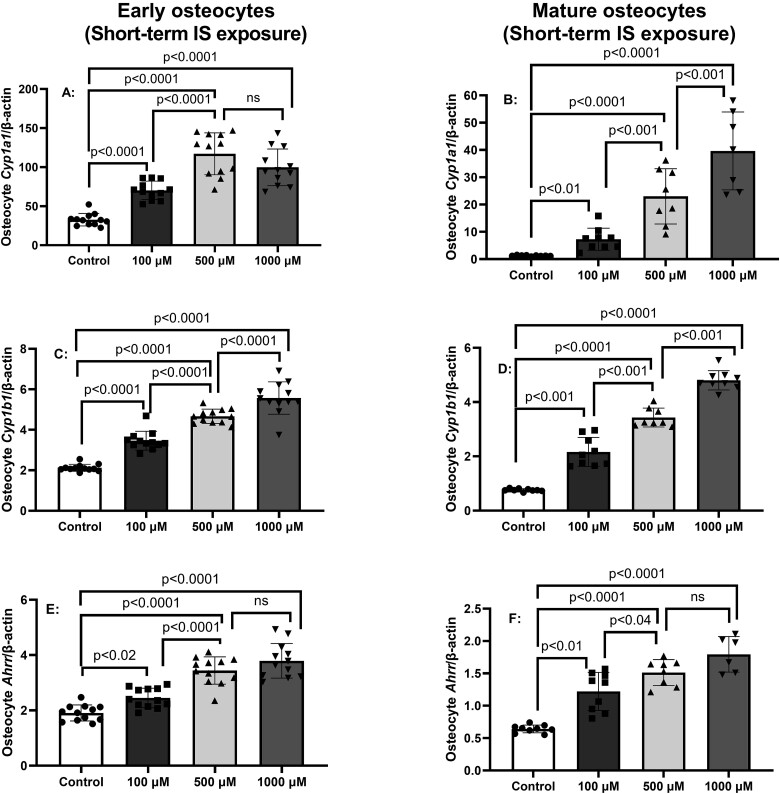
Short-term exposure to indoxyl sulfate (IS) activates aryl hydrocarbon canonical signaling. IDGSW3 osteocytes were cultured for 14 or 35 days (early and mature osteocytes, respectively), then treated for 24 h with 0, 100, 500, or 1000 μM IS. The results in early and mature osteocytes showed a dose-dependent increase in gene expression of AhR activation gene “read out” of Cyp1a1 (A and B), *Cyp1b1* (C and D), and Ahrr (E and F). Data are shown as mean ± SD (*n* = 10-12). One-way ANOVA, and, if *p* < .05, Tukey multiple comparison test between groups with *p* value are shown in the graph.

Indoxyl sulfate is a known endogenous ligand/agonist for AhR.^34^ To confirm IS signals through AhR canonical pathway in the IDG-SW3 osteocytes, early and mature osteocytes were incubated with increasing concentrations of IS for 24 h. Figure 2 demonstrates IS dose-dependently increased expression of AhR downstream target gene products *Cyp1a1* (Figure 2A and B) and *Cyp1b1* (Figure 2C and D) in both early and mature osteocytes. IS also dose-dependently induced mRNA expression of *Ahrr* that negatively feedbacks to limit AhR transcriptional activity (Figure 2E and F).

### Long-term exposure to IS for 14 days leads to altered gene expression and mineralization, with differential signaling through AhR in early osteocytes

**Figure 3:**
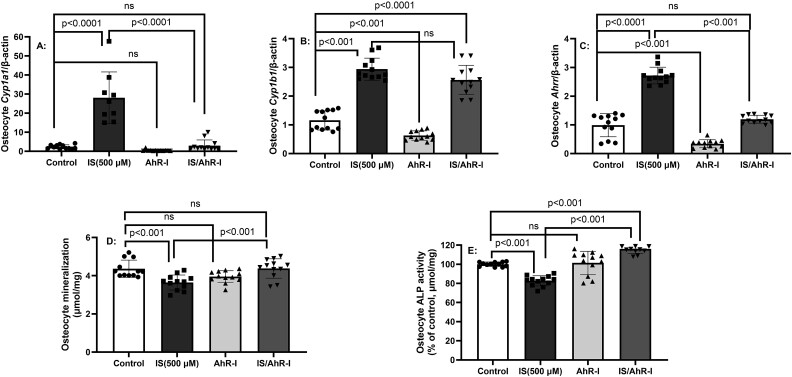
Long-term exposure to indoxyl sulfate (IS) in early osteocytes activates canonical AhR signaling. IDG-SW3 osteocytes were cultured for 14 days with 500 μM IS and gene expression, alkaline phosphatase activity, and mineralization determined in the presence or absence of CH223191, an inhibitor of AhR (labeled AhR-I in figure) that prevents ligand binding and subsequent nuclear translocation of the AhR-ligand complex. The results demonstrate that AhR-I (CH223191) altered ISinduced expression of Cyp1a1 (A) and Ahrr (C) but not *Cyp1b1* (B). AhR-I reversed the IS-induced impairment of mineralization (D) and alkaline phosphatase activity (E). Data are shown as mean ± SD (*n* = 10-12). One-way ANOVA, and, if *p* < .05, Tukey multiple comparison test between groups with *p* value are shown in the graph.

To determine the effect of continuous long-term exposure to IS and confirm a critical role of canonical AhR signaling in IS’s effect on osteocyte gene expression, IDW-SW3 osteocytes were treated with IS (500 μM) in the presence or absence of CH223191 that blocks agonist binding and subsequent nuclear translocation of the AhR-ligand complex^28^^,^^29^ for 14 days, as cells differentiated to early osteocytes. As expected, the 14-day exposure to IS-induced AhR downstream target gene expression of *Cyp1a1*, *Cyp1b1*, and *Ahrr* in osteocytes (Figure 3A-C). Treatment with AhR inhibitor CH223191 (AhR-I) prevented IS-induced increases in *Cyp1a1* and *Ahrr* (Figure 3A and C) but had no effect on *Cyp1b1* (Figure 3B). IS incubation for 14 days inhibited the low levels of mineralization in early osteocytes and reduced ALP activity, and these were reversed with AhR inhibitor (Figure 3D and E) indicating canonical AhR signaling involvement. However, AhR inhibitor CH223191 had no effect on IS-induced mRNA expression of *Sost*, *Dkk1*, *Dmp1*, *Fgf23*, or IS-induced suppression of *Tnfsf11/Tnfrsf11b* ratio (Figure S2). These studies demonstrate that AhR canonical signaling alters ALP and mineralization in early osteocytes, but not genes involved in Wnt inhibition (SOST/Dkk1) or osteoclast activation (RANKL/OPG), indicating IS induces AhR signaling for ALP activity and mineralization but may signal through an alternative pathway for osteocyte gene expression*.*

**Figure 4:**
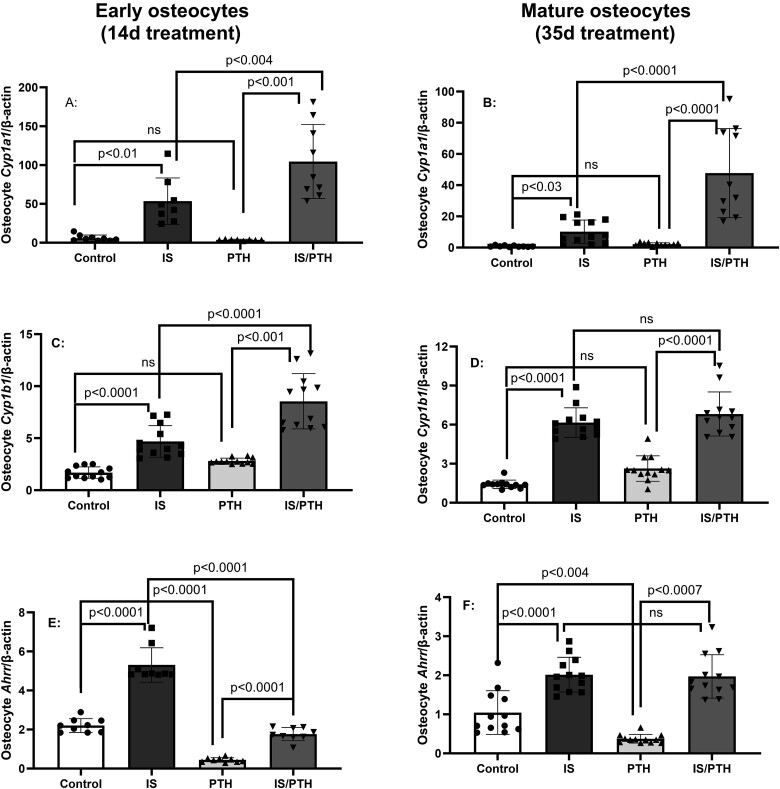
Indoxyl sulfate (IS) and parathyroid hormone (PTH) interact to alter AhR signaling and gene expression in early and mature osteocytes: IDG-SW3 osteocytes were cultured with 500 μM IS in the presence or absence of 100 nM PTH for 14 or 35 days, early and mature osteocytes, respectively. PTH alone had no effect, but was synergistic with IS on the expression of Cyp1a1 (A and B) in early or mature osteocytes and *Cyp1b1* (C and D) in early osteocytes. However, PTH inhibited the positive effects of IS on Ahrr expression, which feeds back to inhibit CYP enzymes, in early osteocytes but not mature osteocytes (E and F). Data are shown as mean ± SD (*n* = 10-12). One-way ANOVA and, if *p* < .05, Tukey multiple comparison test between groups with *p* value are shown in the graph.

### The combination of IS and PTH alters AhR activation, genes for differentiation, mineralization, and osteoclast activation in long-term cultures in both early and mature osteocytes

Because many patients with advanced CKD have both elevated IS and PTH we examined the interaction. IDG-SW3 osteocytes were incubated with osteogenic media and treated with IS (500 μM) in the presence or absence of 100 nM PTH^30^^,^^35^ for 14 or 35 days, during differentiation to early and mature osteocytes, respectively. PTH alone had no effect on *Cyp1a1* or *Cyp1b1* expression early and mature osteocytes but enhanced the IS-induced expression of *Cyp1a1* in both early and mature osteocytes and expression of *Cyp1b1in early osteocytes* (Figure 4A–D). However, PTH inhibited *Ahrr* expression and prevented the IS-induced stimulation of *Ahrr* in early but not late osteocytes, disrupting the normal negative feedback loop activity of Ahrr (Figure 4E and F). PTH alone markedly decreased the expression of *Sost (only in mature osteocytes), Dkk1*, *Fgf23*, and *Dmp1* (in both early and mature osteocytes) and markedly inhibited or completely negated the effects of IS on these same genes in both early and mature osteocytes (Figure S3), indicating the effect of IS is negated in the presence of PTH.

**Figure 5:**
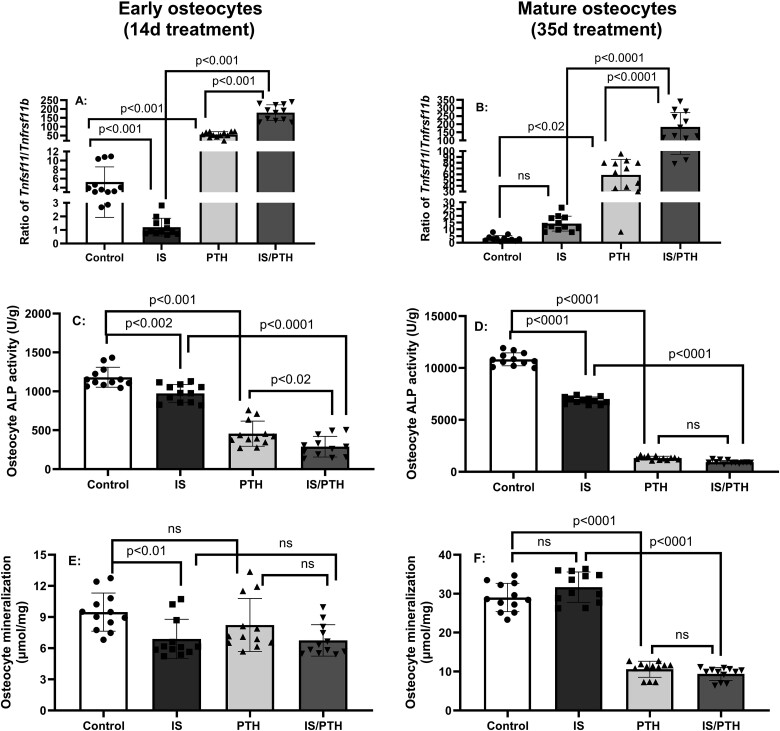
Parathyroid hormone (PTH) and indoxyl sulfate (IS) show stage-dependent interactions in RANKL/OPG ratio, alkaline phosphatase (ALP) activity and mineralization: IDG-SW3 osteocytes were cultured with 500 μM IS in the presence or absence of 100 nM PTH for 14 or 35 days, early and mature osteocytes, respectively. PTH alone increased expression of Tnfsf11/Tnfrsf11b ratio in both early and mature osteocytes; IS alone decreased expression of Tnfsf11/Tnfrsf11b ratio in early but not in mature osteocytes. However, IS in the presence of PTH additively increased expression of Tnfsf11/Tnfrsf11b ratio in both early and mature osteocytes (A and B). Both IS and PTH alone decreased ALP activity in early and mature osteocytes and were additive in reducing ALP activity in early osteocytes (C and D). IS, but not PTH alone, decreased mineralization without additive effects of the combination in early osteocytes (E). However, in mature osteocytes, when mineralization is more pronounced, IS had no effect, but PTH dramatically lowered mineralization, even in the presence of IS (F). Data are shown as mean ± SD (*n* = 10-12). One-way ANOVA and, if *p* < .05, Tukey multiple comparison test between groups with *p* value are shown in the graph.

In contrast, PTH alone increased expression of *Tnfsf11/Tnfrsf11b* ratio in both early and mature osteocytes, while IS alone suppressed the expression of *Tnfsf11/Tnfrsf11b* ratio in early, but had no effect on mature osteocytes (Figure 5A and B). However IS was additive to the stimulatory effect of PTH in increasing osteoclast stimulatory activity, in both early and mature osteocytes, as demonstrated by increased Tnfsf11/Tnfrsf11b ratio (Figure 5A and B). Both IS and PTH alone decreased ALP activity in early and mature osteocytes and were additive in reducing ALP activity in early osteocytes (Figure C and D). IS, but not PTH alone, decreased mineralization without additive effects of the combination in early osteocytes (Figure 5E), a stage with minimal mineralization.^27^ However, in mature osteocytes when mineralization is more pronounced, IS had no effect, but PTH dramatically lowered mineralization, even in the presence of IS (Figure 5F). These results suggest that IS decreases and PTH increases RANKL/OPG ratio, but together are additive. In contrast, there was no interaction of IS and PTH in ALP and mineralization at either stage.

### IS altered PTH1 receptor signaling in early and mature osteocytes

**Figure 6:**
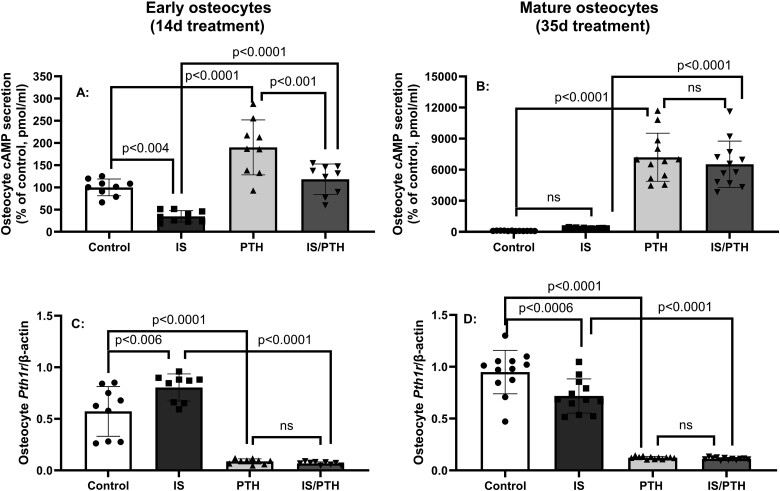
Indoxyl sulfate (IS) altered parathyroid hormone (PTH)1 receptor signaling in early and mature osteocytes: IDG-SW3 osteocytes were cultured with 500 μM IS in the presence or absence of 100 nM PTH for 14 or 35 days, early and mature osteocytes, respectively. In early osteocytes, IS decreased cAMP secretion and reduced the PTH induced cAMP secretion (Figure 6A). However, in mature osteocytes, IS had no effect on cAMP secretion but PTH increased cAMP without an interaction of IS and PTH (Figure 6B). IS increased the Pth1rexpression in early osteocytes (Figure 6C), but decreased the expression in mature osteocytes (Figure 6D). PTH alone decreased the expression of Pth1r in both early and mature osteocytes. Data are shown as mean ± SD (*n* = 10-12). One-way ANOVA and, if *p* < .05, Tukey multiple comparison test between groups with *p* value are shown in the graph.

PTH receptor activation increases cAMP.^36^ To determine if IS alters this signaling pathway as previously reported in osteoblasts,^12^ we cultured the IDG-SW3 osteocytes with IS (500 μM) in the presence or absence of 100 nM PTH for 14 or 35 days and measured cAMP secretion and *Pth1r* expression. In early osteocytes, exposure to IS for 14 days decreased acute cAMP secretion and reduced PTH-induced cAMP secretion (Figure 6A). However, in mature osteocytes, IS had no effect on cAMP secretion but PTH increased cAMP without an interaction of IS and PTH (Figure 6B). IS increased *Pth1r* expression in early osteocytes (Figure 6C), but decreased the expression in mature osteocytes (Figure 6D). As expected, the addition of PTH decreased *Pth1r* expression in both early and mature osteocytes. These data show that IS increases PTH receptor expression but decreases cAMP signaling in early, but not mature osteocytes, but the effect of PTH overrides these changes.

## Discussion

 Advanced kidney disease is associated with multiple comorbidities including a 2- to 5- fold increase in prevalence of hip fractures.^2^ Further, the mortality after a hip fracture is doubled in patients with CKD.^37^ It has long been presumed that the predominant etiology of the increase in fractures is elevated PTH, but fractures occur at both low and high PTH,^4^^,^^5^ and there is evidence for osteoblast differentiation defects earlier in the course of CKD.^6^^,^^7^ In this study, we found a major effect of both short- and long-term exposure of IS on osteocytes, the “master regulator” of bone remodeling. In early and mature osteocytes, short-term exposure of IS for 24 h dose-dependently increased the mRNA expression for the osteocyte Wnt inhibitors *Sost* and *Dkk1* but had variable effects on the expression of *Tnfsf11/Tnfrsf11b* ratio (RANKL/OPG). The short-term exposure also confirmed that IS activated the AHR pathway in both early and mature osteocytes and allowed us to choose the concentration of 500 μM for long-term cultures in the subsequent experiments, mimicking the CKD environment of continuous high exposure of IS in the presence or absence of elevated PTH.

In long-term exposure of IS in osteocytes for 14 or 35 days, decreased expression of *Tnfsf11/Tnfrsf11b* ratio in early but not mature osteocytes and increased mRNA expression for *Sost*/*Dkk1 (*Figure 5 and Figure S3*)*. However, IS interacted with PTH to additively increase the expression of *Tnfsf11/Tnfrsf11b* ratio in both early and mature osteocytes, indicating enhanced osteoclast activity with IS in the presence or absence of PTH. The results also demonstrated IS in the absence of PTH, increased osteocyte mRNA *Wnt* inhibitor expression in both early and mature osteocytes, and decreased mineralization albeit only in early osteocytes when mineralization is limited. These results support that the elevations of IS in patients with CKD, due to both increased production and decreased excretion, may explain the decreased bone remodeling in early CKD when PTH is still relatively normal and in late CKD when PTH is lower or “over suppressed” by therapies. These results also explain our previous study in the Cy/+_IU_ rat model of progressive CKD-MBD where anti-sclerostin antibody increased trabecular bone volume/total volume (BV/TV) and trabecular mineralization surface in animals with low PTH, but not in animals with high PTH as SOST was already suppressed.^38^ These data also explain why suppressing PTH as a treatment approach may not always improve fracture risk due to the underlying changes induced by continuously elevated IS in CKD.

Previous studies have found that IS had a dose-dependent effect to inhibit primary mouse osteoblast gene expression (RUNX2, OSX, BMP2, Col1A1, ALP) in primary osteoblasts^39^ and mesenchymal stem cells.^11^ In the only other study of IS in osteocytes, IS dose-dependently decreased DMP1 protein (the only measured outcome) in MLO-Y4 cells.^40^ With long-term exposure to IS, *Dmp1* mRNA expression was increased in early but not changed in mature osteocytes. In parallel, long-term exposure of IS increased *Fgf23* in both early and mature osteocytes. These data are inconsistent with *in vivo* data in CKD animals demonstrating downregulation of DMP1 associated with osteocyte abnormalities, including impaired mineralization, that are improved with DMP1 administration and lower FGF23,^41^ possibly reflecting *in vitro* versus *in vivo* conditions. As expected, PTH markedly reduced *Dmp1* expression, suggesting that PTH is more likely than IS to affect FGF23/DMP1, at least in vitro.

Indoxyl sulfate is a known potent ligand of the AhR receptor,^34^ involved in binding and metabolizing exogenous and endogenous toxins. The receptor is colocalized in the cytoplasm in a multiprotein complex. Once ligands bind, the AhR complex disassociates and dimerizes with the AhR nuclear translocator. The dimer then binds to the xenobiotic-responsive element, leading to P450 *CYP1A1*, *CYP1A2*, and *CYP1B1* metabolizing enzymes that oxidize the bound toxin to increase polarity of the ligand and enable metabolism, so called canonical signaling.^42^ In patients with CKD and in 5/6 nephrectomized mice, there is activation of AhR,^43^ which, based on our data, may be due to the high levels of IS. Our studies found that blocking agonist IS binding and subsequent nuclear translocation of AhR with CH223191 in the IDG-SW3 osteocytes decreased IS-induced expression of *Cyp1a1* and *Ahrr*, but not *Cyp1b1* expression, indicating some inhibition of canonical signaling. However, CH223191 predominately affected mineralization and ALP activity, but not mRNA expression of *Sost/Dkk1 or the Tnfsf11/Tnfrsf11b expression* ratio in osteocytes. However, the net effect of AhR is related to nuclear coactivators, leading to a variety of downstream signaling interactions including nuclear factor kappa beta (NFκβ) and tissue factors such as Krüppel-like factor 6 (KLF6).^42^ In addition, the AhR has effects on other signaling pathways including MAPK pathway and ubiquitination-degradation.^44^ Furthermore, there is known epigenetic alterations that can differentially affect *Cyp* gene expression,^45^ and other regulators including cAMP,^46^ which might explain the differential effect of PTH on AHRR. Taken together, the IS-induced changes in osteocyte expression of *Sost/Dkk1/Tnfsf11/Tnfrsf11b* ratio may still be dependent on the AhR despite the lack of effect of CH223191, and further deciphering these signaling pathways will require transgenic approaches.

These studies highlight the complexity of bone in CKD and in particular the importance of non-PTH uremic toxins on osteocytes. Our work utilized IDG-SW3 osteocytes that differentiate in culture to mature osteocytes, which were chosen because this cell line is the only known line that express FGF23, another important uremic toxin. Despite our novel findings, our study had limitations. Although an ideal cell line, these in vitro studies must be confirmed in vivo and studies are in progress. While we studied multiple doses of IS ultimately choosing 500 μM for long-term cultures, we chose to focus only on 1 dose of PTH that is frequently used in cultures^30^ but may not reflect the very high levels in CKD or, conversely, the normal oscillations of PTH in the absence of CKD. Finally, we used CH223191 as a known compound to alter 1 signaling pathway of AhR but further work is required to elucidate other signaling pathways as there is no perfect inhibitor for *in vitro* use. At the present time, the only way to lower IS is through intestinal “detoxins” such as AST-120 that suppresses low turnover progression in CKD rats,^47^ and we have shown the administration of dietary fiber inulin to our Cy/+ rat^48^ also reduced IS and reduced bone turnover through reduction in osteoclast activity. At this time, the intestine may be superior to control IS in CKD as opposed to dialysis due to the significant protein binding of IS rendering it unremovable by dialysis.

In summary, IS increases osteocyte Wnt inhibitor expression, which would inhibit differentiation/bone formation and expression of *Fgf23* and *Dmp1*, with opposite effects in the presence of PTH. Indeed, IS interacted with PTH to additively increase the expression of *Tnfsf11/Tnfrsf11b* ratio, which would enhance bone resorption. IS clearly signals through the AhR canonical signaling pathway but it appears likely that other pathways are also involved or there are additional coactivators/repressors of AhR. Further studies are needed to confirm these results *in vivo,* to directly confirm the role of AhR. Importantly, understanding this novel mechanism of bone loss in CKD may help explain the abnormal bone at low levels of PTH, leading to CKD-specific treatment options to prevent bone fractures, a major cause of morbidity and mortality in CKD.

## Supplementary Material

Supplemental_Figures_ziae136

## Data Availability

Original data are available upon request from the corresponding author after publication.
