# Supplementary material for: The uremic toxin indoxyl sulfate decreases osteocyte RANKL/OPG and increases Wnt inhibitor RNA expression that is reversed by PTH
Source: JBMR Plus. 2024 Oct 29;9(1):ziae136. doi: 10.1093/jbmrpl/ziae136 (PMC11631378; doi:10.1093/jbmrpl/ziae136)
Supplement: Supplemental_Figures_ziae136 [file supplemental_figures_ziae136.docx]

**Supplemental Figures**

**
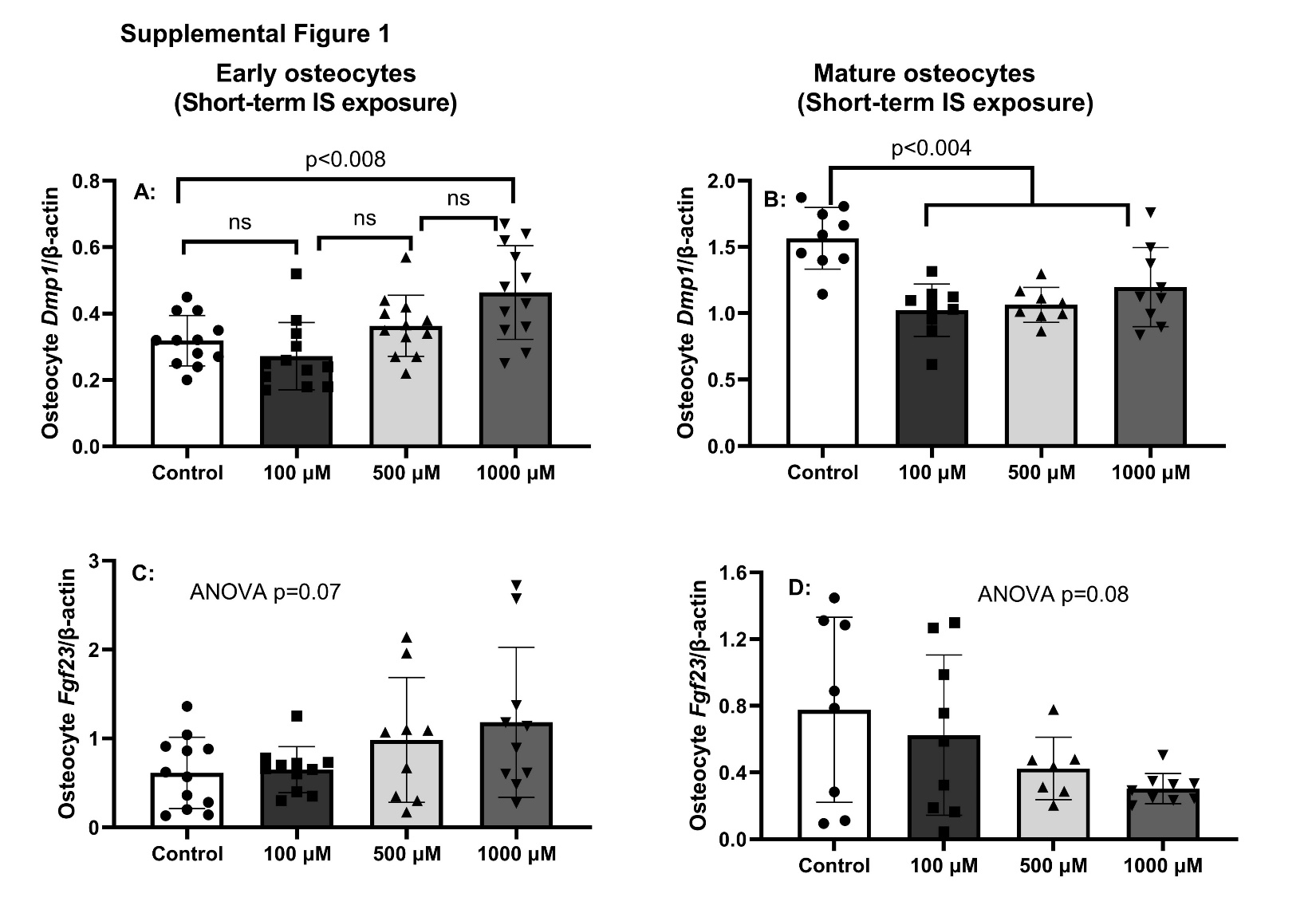
**

**Supplemental Figure 1:** **Short-term exposure to indoxyl sulfate (IS) has minimal effect on mineralization genes.** IDG-SW3 osteocytes were cultured for 14 or 35 days (early and mature osteocytes, respectively), then treated for 24 hours with 0, 100, 500 or 1000 µM indoxyl sulfate. The results demonstrate that in early osteocytes only the highest concentration of IS increases *Dmp1* expression (A), and in mature osteocytes all three tested concentrations lower DMP1 expression (B). There was no effect of short-term exposure of IS on *Fgf23* expression in early (C) or mature (D) osteocytes. Data are shown as mean ± SD (n =10-12). One-Way ANOVA, and if p < 0.05, Tukey’s multiple comparison test between groups with p value shown in graph.

**Supplemental Figure 2: Inhibition of AhR activity had no effect on indoxyl sulfate-induced osteocyte gene expression in early osteocytes.** IDG-SW3 osteocytes were cultured for 14 days with 500 µM indoxyl sulfate (IS) in the presence or absence of CH223191, an inhibitor of AhR (AhR-I) that prevents ligand binding and subsequent nuclear translocation of the AhR-ligand complex, and gene expression determined. The results demonstrate that AhR-I (CH223191) had no effect on IS-induced expression of *Sost* (A), *Dkk1* (B), *Dmp1*(C), *Fgf23* (D) and IS-induced suppression of *Tnfsf11/Tnfrsf11b* ratio (E). This suggests alternate signaling may predominate. Data are shown as mean ± SD (n =10-12). One-Way ANOVA, and if p < 0.05, Tukey’s multiple comparison test between groups with p value shown in graph.

**
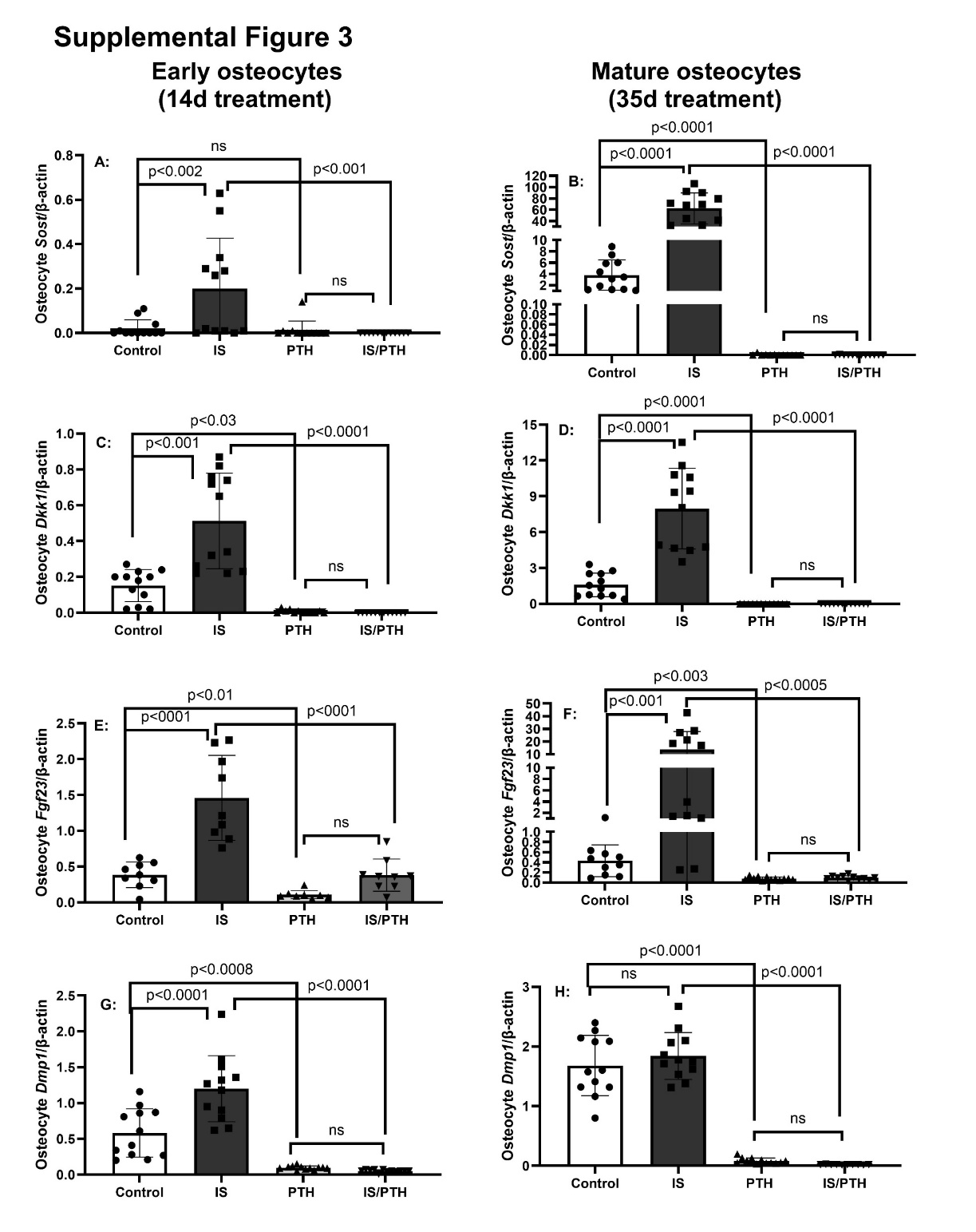
**

**Supplemental Figure 3:** **PTH negates indoxyl sulfate-induced gene expression in early and mature osteocytes:** IDG-SW3 osteocytes were cultured with 500 µM indoxyl sulfate (IS) in the presence or absence of 100 nM PTH for 14 or 35 days, early and mature osteocytes, respectively. PTH alone decreased or had no effect on the expression of *Sost* (A and B), *Dkk1* (C and D), *Fgf23* (E and F), and *Dmp1* (G and H) and markedly inhibited or completely negated IS- induced change in these same genes in both early and mature osteocytes. Data are shown as mean ± SD (n =10-12). One-Way ANOVA, and if p < 0.05, Tukey’s multiple comparison test between groups with p value shown in graph.
